# The cell death regulator c-FLIP_R_ impairs natural killer cell responses during influenza a virus infection

**DOI:** 10.1007/s00109-025-02627-9

**Published:** 2025-12-26

**Authors:** André Carmo-Fernandes, Neda Tafrishi, Clara Bessen, Konstantinos Katsoulis-Dimitriou, Marina C. Greweling-Pils, Dunja Bruder, Carlos Plaza-Sirvent, Ingo Schmitz

**Affiliations:** 1 Department of Molecular Immunology, Medical Faculty, Ruhr University Bochum, Universitaetsstrasse 150, 44801 Bochum, Germany; 2 Systems-Oriented Immunology and Inflammation Research Group, Helmholtz Centre for Infection Research, Brunswick, Germany; 3 Institute for Molecular and Clinical Immunology, Medical Faculty, Otto-Von-Guericke-University, Magdeburg, Germany; 4 Core Facility of Comparative Medicine, Helmholtz Centre for Infection Research, Brunswick, Germany; 5 Infection Immunology Group, Institute of Medical Microbiology and Hospital Hygiene, Otto Von Guericke University Magdeburg, Magdeburg, Germany; 6 Immune Regulation Group, Helmholtz Centre for Infection Research, Brunswick, Germany

**Keywords:** Apoptosis, c-FLIP, Influenza A virus, Natural killer cells

## Abstract

**Abstract:**

Apoptosis, a form of programmed cell death, is crucial for keeping homeostasis during and after infections. Cellular FLICE-inhibitory protein (c-FLIP) is an inhibitor of death receptor-mediated apoptosis, of which three isoforms have been characterized so far. While the isoforms c-FLIP_long_ and c-FLIP_short_ are well characterized, the function of c-FLIP_R_ remains poorly understood. To study the role of c-FLIP_R_ in influenza A virus (IAV) infection, we employed vavFLIP_R_ transgenic mice that constitutively express murine c-FLIP_R_ in all hematopoietic cells. Upon IAV challenge, vavFLIP_R_ mice showed an altered viral dynamic with a higher viral load than wild-type mice, coinciding with a higher number of Natural Killer (NK) cells. IAV directly infected murine NK cells, but viral particles produced by NK cells did not infect other target cells. While NK cells from vavFLIP_R_ and control mice were equally able to kill tumor cells in vitro, we detected reduced degranulation of c-FLIP_R_ transgenic NK cells from infected mice compared to wild-type counterparts. Furthermore, TNFα and IFNg expression was reduced in c-FLIP_R_ transgenic NK cells. We conclude that c-FLIP_R_ impairs NK cell activity during IAV infection.

**Key messages:**

- Constitutive expression of the anti-apoptotic c-FLIP_R_ in hematopoietic cells increases IAV titers.
- Accumulation of NK cells in vavFLIP_R_ mice during IAV infection.
- IAV infects NK cells in a non-productive manner.
- IAV infection of NK cells impairs their function.

## Introduction

Apoptosis is a form of programmed cell death, mainly characterized by cell size reduction, fragmentation of the cytoskeleton, and formation of vesicles—called apoptotic bodies—which are phagocytosed afterward [1]. During a typical immune response, apoptosis is critical in controlling infections by limiting resources available for pathogen replication [2, 3]. Moreover, apoptosis is crucial to regulate immune responses during and after infection, as it maintains the number of immune cells at a reasonable level to prevent autoimmune responses. Next to apoptosis, different forms of lytic programmed cell death have been described, including necroptosis and pyroptosis, which depend on RIP kinases and inflammasomes, respectively [4]. Lytic cell death forms are pro-inflammatory and help to activate the immune system during infection [4].

Although many aspects of apoptosis have been identified, regulation of apoptosis, especially by c-FLIP proteins, remains a matter of interest in experimental research [5–8]. c-FLIP proteins act at the level of the CD95 death-inducing signaling complex (DISC), and c-FLIP_long_ and c-FLIP_short_ inhibit caspase-8 activation via two different mechanisms [6, 9]. Further studies on c-FLIP protein expression in cell lines of various species demonstrated that only humans are able to express c-FLIP_short_, while the only short c-FLIP isoform that other species can express is c-FLIP_R_ [10, 11]. However, the function of c-FLIP_R_ in the immune system is still poorly understood.

Using vavFLIP_R_ transgenic mice, in which c-FLIP_R_ is constitutively expressed in all hematopoietic cells, we previously investigated the role of c-FLIP_R_ in activated lymphocytes [8]. For this purpose, vavFLIP_R_ mice were challenged with *Listeria monocytogenes*. Of note, vavFLIP_R_ mice exhibited better bacterial clearance, less liver necrosis, and less caspase-3 activation compared to infected wild-type (WT) mice. These observations suggested that early apoptosis during *Listeria monocytogenes* infection may restrict the bacterial clearance by CD8^+^ cytotoxic T-lymphocytes (CTLs) [8].

Influenza A virus (IAV) remains an ongoing worldwide threat for public health [12–14] and understanding the link between regulatory mechanisms such as cell death, immune, and viral dynamics may facilitate the development of novel preventive and treatment strategies. IAV is well known to induce apoptosis in human cell lines [15]. However, studies using gene-modified mice demonstrated that necrotic forms of cell death are also induced by IAV in vivo. In that respect, mice deficient in *Ripk3*, a central mediator of inflammation and necroptosis, are more susceptible to IAV infection, which was attributed to both MLKL-driven necroptosis and FADD-mediated apoptosis [16]. The contribution of necroptosis may depend on the viral dosage and additional factors [17, 18]. Upstream of RIPK3 is the nucleic acid sensor ZBP1 (aka DAI), which detects Z-RNA that occurs during IAV infection [19–21]. Moreover, the NLRP3 inflammasome has been shown to contribute to IAV detection and the production of IL-1 family cytokines [22]. Since inflammasome-activated caspase-1 can lead to pyroptosis, IAV may also induce pyroptosis [23] or PANoptosis, a combination of pyroptosis, apoptosis, and necroptosis [24].

While at later stages of IAV infection, i.e., when adaptive immunity is activated, the virus is eradicated by CTLs; the early phase of the anti-viral immune response is believed to rely on Natural Killer (NK) cells. However, the exact role of NK cells during IAV infection is not clear, and both protective and immunopathogenic roles have been reported. On the one hand, NK cells directly kill infected target cells via the perforin/granzyme and death ligand pathways [25] and secrete cytokines to recruit further immune cells [26]. On the other hand, NK cells can exacerbate immune cell-mediated pathology during IAV infection [27].

In this study, we aimed to elucidate the role of c-FLIP_R_ in antiviral immunity by examining the consequences of constitutive c-FLIP_R_ expression on the immune response against IAV infection. In contrast to the improved bacterial clearance observed in the *Listeria* model [8], vavFLIP_R_ mice showed a higher viral load compared to control mice when challenged with IAV. Analysis of immune cells during IAV infection indicated that although the frequency and the absolute cell number of most of the immune cells were similar, vavFLIP_R_ mice showed a higher number of NK cells at the peak of infection. We found non-productive infection of NK cells and reduced degranulation of c-FLIP_R_-expressing NK cells, suggesting impaired function of c-FLIP_R_ transgenic NK cells.

## Materials and methods

### Mice

The mouse strain vavFLIP_R_ was previously described and expresses murine c-FLIP_R_ cDNA in all hematopoietic cells under the control of the *vav* promoter [8, 28]. Mice carrying the transgenic construct were backcrossed for more than ten generations to C57BL/6 mice. Genotyping was performed by PCR on tail biopsies using KAPA Mouse Genotyping Hot Start Kit (PEQLAB) according to the manufacturer’s protocol. All mice were kept under specific pathogen-free (SPF) conditions in the animal facilities of the Helmholtz Centre for Infection Research, Braunschweig, and the Medical Faculty of the Ruhr University, Bochum, with access to water and food ad libitum. Experimental procedures were approved by the relevant animal experimentation committees and performed in compliance with international and local animal welfare legislations (Niedersächsisches Landesamt für Verbraucherschutz und Lebensmittelsicherheit and Landesamtes für Natur, Umwelt und Verbraucherschutz Nordrhein-Westfalen).

### Preparation of influenza A virus

Influenza A (PR8, A/Puerto Rico/8/34 H1N1) viral stocks were propagated in MDCK cells. Briefly, MDCK cells were incubated in DMEM with 10% FCS, 5% L-glutamine, 1% penicillin/streptomycin, and 1% non-essential amino acids at 37 °C in a 5% CO_2_ incubator for 1–2 days. The growth medium was replaced with infection medium (MEM containing 2.5 µg/mL N-Acetylated Trypsin, Sigma, 0.1% bovine serum albumin (BSA), 1% penicillin/streptomycin). The infection medium was decanted and washed three times with 10 mL DPBS. DPBS was removed from the flask with a sterile pipette. 5 µL virus stock (PR8 in egg allantoic fluid) was inoculated in 20 mL infection media to each flask using sterile pipettes, including positive and negative controls. After 1 h of incubation at 37 °C in a 5% CO_2_ incubator, the virus suspension was removed, and the cells were washed once with 10 mL DPBS (0.2% BSA). Afterward, 30 mL of the infection culture media was added to each flask. Cells were checked for clear cytopathic effects in the virus-infected flasks (otherwise, waited another 12 or 24 h). When cytopathic effects were observed, the supernatant was collected and centrifuged to remove debris. Small aliquots of the virus were kept at −80 °C until further use. Viral titer was determined by the focus-forming unit (FFU) assay.

### Flow cytometry

For flow cytometry analysis, 1 million cells were washed in 1 mL PBS at 4 °C, 450 g, 5 min. Cells were resuspended in 100 μL PBS and stained with LIVE/DEAD fixable blue stain (Invitrogen) for 30 min, 4 °C in the dark. Thereafter, cells were washed in 1 mL PBS as above. Next, cells were resuspended in FACS buffer containing Fc Block antibodies (TruStain FcX™, # 101320, Biolegend) and incubated for 15 min at 4 °C in the dark. Surface markers were stained in 100 μL FACS buffer. Cells were incubated with appropriate dilutions of fluorochrome-conjugated antibodies at 4 °C for 20 min in the dark. Unbound antibodies were washed away with 1 mL FACS buffer. Labeled samples were analyzed on BD LSR Fortessa and BD LSR II by Becton Dickinson (New Jersey, USA). Cell purification was performed using FACS Aria II (Becton Dickinson) or Moflo (Beckman Coulter, Indianapolis, USA) devices. Antibodies used were CD3-Horizon V500 (500A2, BD Biosciences), CD4-PacificBlue (L3T4, Biolegend), CD8-PECy7 (53-6.7.7.7.7, Biolegend), CD11b-BV785 (M1/70, Biolegend), CD19-PerCPCy5.5 (eBio1D3, eBioscience), CD19-APCCy7 (6D5, Biolegend), CD27-BV650 (LG.3A10, Biolegend), CD107a-BV421 (1D4B, Biolegend), Granzyme B-Pacific Blue (GB11, Biolegend), IFNγ-PECy7 (XMG1.2, eBioscience), KRLG1-PECy7 (2F1/KLRG, Biolegend), NK1.1-PE (PK136, BD Biosciences), NKp46-eFluor660 (29A1.4, eBioscience), TNFα-PE (MP6-XT22, Biolegend), and Influenza NP 311–325 Tetramer-APC (# TS-M716-2, MBL). Apoptosis was analyzed by staining with CellEvent Caspase 3/7 Green (Life Technologies) and with Annexin V-FITC (Biolegend) plus 7-AAD (Enzo) in Binding Buffer (10 mM HEPES/KOH, pH = 7.4, 140 mM NaCl, 2.5 mM CaCl_2_).

### Infection of cells

For infections where the determination of infectious virus was not required, no trypsin was added to the medium. NK cells were purified from single-cell suspensions of splenocytes and sorted with an NK cell isolation kit (Miltenyi Biotec), where all cells except NK cells were magnetically labeled and depleted from the cell suspension using an LS magnetic column (Miltenyi Biotec). MACS-sorted immune cells were infected with virus diluted in serum-free culture medium at an appropriate multiplicity of infection (MOI). Cells were washed before infection in PBS to remove all traces of serum. After an adsorption period of 1 h on a rocking platform at 37 °C, the virus inoculum (or medium only for mock infections) was removed and replaced with serum-free medium. Cells were incubated at 37 °C, 5% CO_2_ until harvested.

For analysis of the productivity of IAV replication in NK cells, magnetically isolated NK cells from transgenic and control mice were pre-activated for 2 h with 50 ng/mL IL-2 and 25 ng/mL IL-15 in RPMI 1640 medium containing 10% FBS, 1% MEM non-essential amino acids (Gibco), 1 mM sodium pyruvate (Gibco), 1% GlutaMAX (Gibco), and 1% penicillin/streptomycin. After pre-activation, viral adsorption was performed for 2 h at 37 °C, 5% CO_2_. NK cells were then washed five times with PBS to remove extracellular viral particles and incubated for 16 and 24 h in fresh medium containing IL-2 and IL-15 for virus production. After the incubation period, the supernatant was collected, and a serial dilution of 10, 20, 50, or 100 µL of supernatant was transferred to MDCK cells. MDCK cells were incubated in DMEM (high glucose) with 10% FBS and 1% penicillin/streptomycin for 24 h with NK cells’ supernatant or infected with virus with the corresponding MOI as a positive control. NK cells, NK cells’ supernatant (20 µL), and PBS-washed MDCK cells were processed for RNA isolation to determine viral load by RT-qPCR.

An aliquot of NK cells after 16- and 24-h virus production was obtained and directly stained with 7-AAD for 10 min for flow cytometry analysis to evaluate NK cell viability after infection and virus production.

### In vivo infection with IAV

Female animals (12–17 weeks of age) were anesthetized by intraperitoneal injection of Ketamine-Xylazine solution (10% (v/v) 100 mg/ml ketamine and 5% (v/v) 20 mg/mL xylazine in 0.9% (w/v) NaCl) with a dose adjusted to the individual body weight (10 µL per g body weight) and infected intranasally with 2 × 10^3^ FFU IAV in 20 µL sterile PBS. Weight loss and survival of infected mice were monitored over a 14-day period. Animals showing more than 20% of body weight loss were euthanized and documented as dead. Mice were evaluated daily and scored for individual symptoms. Ruffled fur (absent = 0; present = 1), hunched back (absent = 0; present = 1), and activity (normal = 0; reduced = 1; severely reduced = 2) were evaluated. The final score was the addition of each individual symptom score. The minimum score was 0 for the control mice and 1–4 for the IAV-infected mice, depending on the infection severity.

### RT-qPCR analyses

For the analysis of *Cflar* (c-FLIP) expression in NK cells, CD3 and CD19-negative, NKp46-positive cells were purified by flow cytometry and stimulated for 3 h with 50 ng/mL murine interleukin-2. Subsequently, RNA was isolated using the RNeasy Mini Kit (Qiagen), including an on-column genomic DNA digestion step (Qiagen’s instruction). For the determination of viral load, total RNA from the lung was prepared according to the detailed protocol from the RNeasy Mini Kit (Qiagen), including an on-column genomic DNA digestion step (Qiagen’s instruction). The concentration of the isolated RNA was measured by Nanodrop 2000c (Thermo Scientific). One hundred nanograms of purified RNA was used as a template to generate a complementary DNA (cDNA) strand by means of the Revert™ Premium First Strand cDNA Synthesis Kit (Thermo Scientific). NP primers were used for RNA transcription according to the supplier’s protocol. cDNA was used as a template for real-time PCR using SYBR Green (Roche). Ubiquitin-conjugating enzyme E2D 2 A (UBC) was used as a housekeeping gene for normalization. qPCR was implemented in a Roche LightCycler® Real-Time device using Roche FastStart SYBRgreen Master (Roche, #04673484001). Measurements were run in duplicates in the LightCycler 96 system using the following primers (NP) 5′-GAG GGG TGA GAA TGG ACG AAA AAC and 3′-CAG GCA GGC AGG CAG GAC TT; (FLIP) 5′-ACC CTC ACC TGG TTT CTGA TT-3′and 5′-TCG TTC TGA TCT AAG CTC TCA CC-3′; (FLIPL) 5′-GCA GAA GCU CUC CCA GCA-3′ and 5′-UUU GUC CAU GAG UUC AAC GUG-3′; (FLIPR) 5′-UCC AGA AGU ACA CCC AGU CCA-3′ and 5′-CAC UGG CUC CAG ACU CAC C-3′; (UBE2D) 5′-AAG AGA ATC CAC AAG GAA TTG AAT G-3′ and 5′-CAA CAG GAC CTG AAC ACT G-3′. Incubation was done in a peqSTAR thermocycler.

### Focus forming assay

Lungs from infected mice were collected at different time points p.i. and homogenized in PBS + 0.1% BSA. Virus titers in these homogenates were determined by the FFU assay on Madin–Darby Canine Kidney (MDCK) cells. Briefly, 6 × 10^4^ MDCK cells per well were seeded in 96-well culture plates and incubated at 37 °C and 5% CO_2_ for 24 h. Serial tenfold dilutions of extracts were prepared in MEM containing 2.5 μg/mL NAT (N-Acetylated Trypsin, Sigma), 0.1% BSA, 1% penicillin/streptomycin (infection medium) and added to MDCK cells. Specific virus stocks (virus in embryonated chicken eggs) were used as positive controls for the assay. After 1 h of incubation at 37 °C in 5% CO_2_, the inoculates were replaced with 100 μL of 1% Avicel overlay prepared in DMEM containing 2.5% NAT, 0.1% BSA, and the cells were incubated for 24 h. Subsequently, the cells were washed twice with PBS and fixed with 4% formaldehyde in PBS (100 μL/well) for 10 min at room temperature. Then, the cells were washed twice and incubated with a quencher (100 μL/well; 0.5% Triton X-100, 20 mM Glycine in PBS) for 10 min at room temperature. Afterward, the cells were washed with Washing Buffer (100 μL/well, 0.5% Tween20 in PBS). The primary antibody (anti-influenza Nucleocapsid NP polyclonal goat antibody, Virostat) and the secondary antibody (anti-goat-HRP from KPL, MA, USA) were diluted 1:1000 in Blocking Buffer (50 μL/well; 0.5% Tween, 20% BSA in PBS). Fifty microliters of the primary antibody was added to each well and incubated at room temperature. After 45 min, the cells were washed with Washing Buffer, incubated with 50 μL of secondary antibody for 45 min, then washed again and incubated with 30 μL of True Blue substrate and exposed until blue spots from infected cells appeared (about 30 min). Foci were counted by microscopy, and viral titers were calculated as focus formation units (FFU) per lung.

### Histology and immunohistochemistry

Mice were sacrificed and lungs were isolated. Samples were fixed with 4% formaldehyde and embedded in paraffin. Three-micrometer sections were cut, deparaffinized, and stained with hematoxylin and eosin for histological analysis. Furthermore, they were stained with NP antibodies. Histological analysis and immunohistochemistry determinations were done at the mouse pathology facility at the Helmholtz Center for Infection Research.

### In vivo NK cell depletion

In order to deplete NK cells in mice with influenza infection, mice were i.p. injected with 50 μg anti-Asialo-GM1 (Affymetrix) every 3 days, starting on the day before infection. Control mice were treated with an equal amount of polyclonal rabbit IgG (Sigma-Aldrich). After depletion treatments, NK cells in the lung and spleen were examined by flow cytometry on day 7.

### Statistical analysis

Statistical significance was calculated by Mann–Whitney test or Kruskal–Wallis one-way ANOVA (Dunn’s multiple comparison post-test) using Prism Software (GraphPad Software, La Jolla, CA). P values were considered significant as follows: *p < 0.05, **p < 0.01, ***p < 0.001, and ****p < 0.0001.

## Results

### *vavFLIP*_*R*_* mice show a higher viral load than WT mice during influenza A infection*

**Fig. 1:**
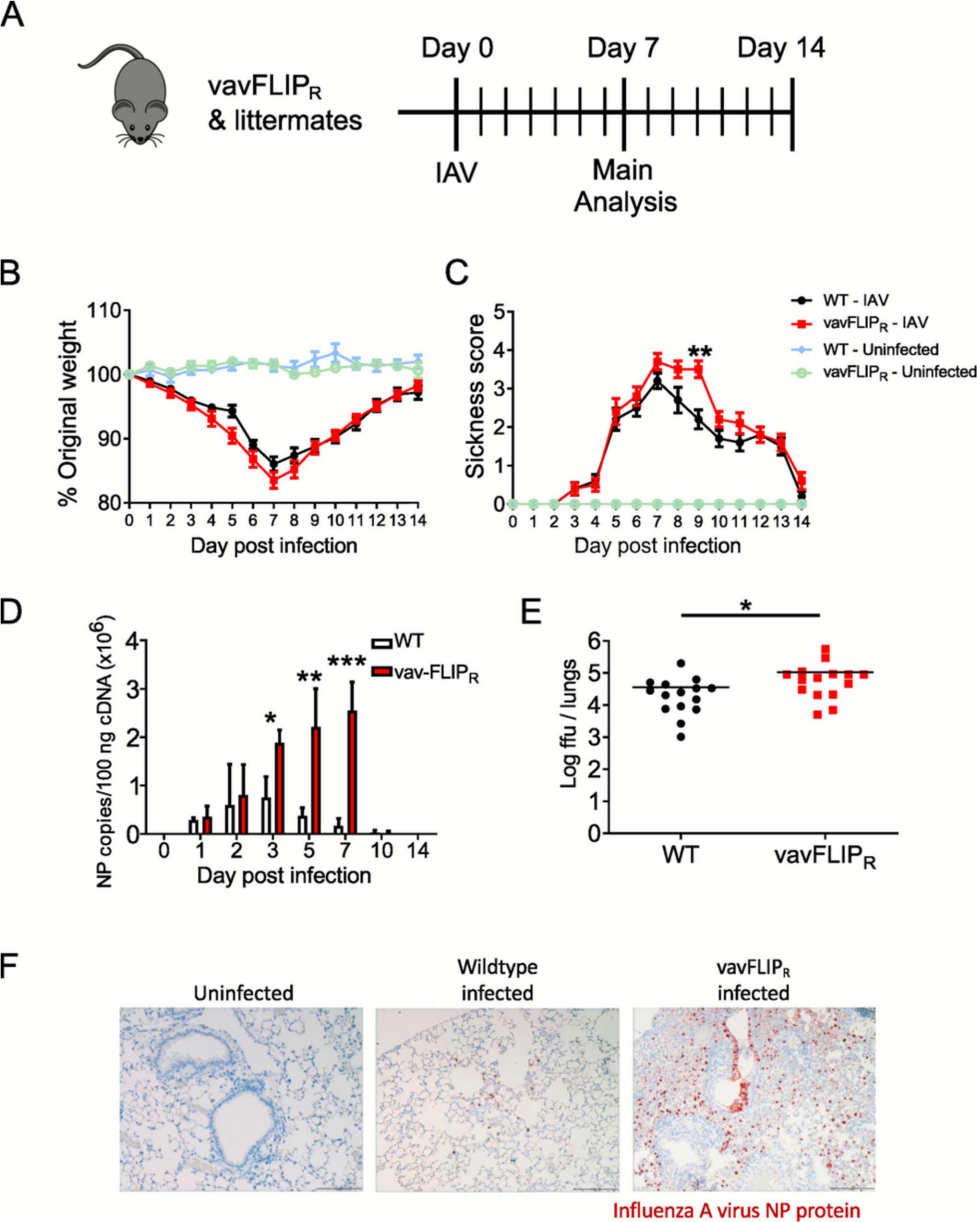
vavFLIP_R_ mice have higher viral loads during influenza infection. **A** Scheme illustrating the experimental setup of influenza A virus (IAV) infection of mice and subsequent analysis. **B** Weight loss comparison during IAV infection. vavFLIP_R_ and WT female mice at the age of 12–17 weeks were infected intranasally with 2 × 10^3^ FFU PR8 (H1N1) influenza A virus. Following infection, mice were checked daily for weight loss until day 14 p.i. Data are presented as mean value ± SEM. **C** Sickness score comparison during IAV infection. Mice were evaluated daily and scored for individual symptoms for 14 days. Ruffled fur (absent = 0; present = 1), hunched back (absent = 0; present = 1) and activity (normal = 0; reduced = 1; severely reduced = 2) were evaluated. The final score was the addition of each individual symptom score. The minimum score was 0 for a control mouse and 1–4 for IAV-infected mice, depending on severity. Data are presented as mean value ± SEM. Statistical analyses were performed using two-tailed Mann–Whitney tests; **p < 0.01. **D** Viral load kinetics in the lungs of the vavFLIP_R_ mice and WT littermates after intranasal infection of female mice at the age of 12–17 weeks with 2 × 10^3^ FFU PR8. Influenza virus titers were evaluated in lung homogenates of WT (white bars) and vavFLIP_R_ (red bars) mice by qPCR with at least two technical replicates for each sample. Bar graphs represent the mean; error bars represent SEM. Statistical analyses were performed using two-tailed Mann–Whitney tests; *p < 0.05, **p < 0.01, and ***p < 0.001. **E** Viral load in the lungs of influenza-infected vavFLIP_R_ (*n* = 5) and WT (*n* = 5) mice were measured on day 7 p.i. by focus formation assay indicating replication-competent virus. Foci assay was performed with three technical replicates for each sample. Statistical analyses were performed using two-tailed Mann–Whitney tests; *p < 0.05. **F** Histological sections of lungs of WT and vavFLIP_R_ mice infected with the PR8 virus. Mice were infected intranasally with 2 × 10^3^ FFU of IAV virus. Control mice were given an equal volume of PBS for mock infection. 7 days post-infection, lung sections were stained with hematoxylin and eosin for histological analysis. Furthermore, they were stained with anti-influenza NP antibody (brown staining)

First, we verified that the inhibition of death receptor-induced apoptosis ensues no alterations in lymphocyte numbers; lymphocyte populations in the spleen and lymph nodes of 12- to 17-week-old vavFLIP_R_ (*n* = 6) and WT littermates (*n* = 6) were analyzed. Consistent with previous studies [8], frequencies and total cell numbers of CD3^+^, CD4^+^, and CD8^+^ T cells, CD19^+^ B cells, and NKp46^+^ cells were comparable between both mouse groups (Table 1). Next, vavFLIP_R_ mice were challenged with IAV to evaluate whether the expression of c-FLIP_R_ modulates the immune response during viral infection. An infection dose of 2 × 10^3^ FFU PR8 (H1N1) was intranasally applied to vavFLIP_R_ and WT mice; control mice were given an equal volume of PBS for mock infection. While most analyses were done at day 7, the peak of infection, body weight and disease scores were recorded daily for 14 days p.i. (Fig. 1A). The weight loss in vavFLIP_R_ and WT mice was largely similar in the early phase of infection. While vavFLIP_R_ mice showed slightly higher body weight loss compared to WT mice at the peak of infection, both types of mice were able to recover and regain body weight (Fig. 1B). Importantly, vavFLIP_R_ mice had a higher sickness score at day 9 p.i. and eventually recovered similarly to WT mice (Fig. 1C).

**Table 1:** Percentage and absolute cell number of cells vavFLIP_R_ and WT littermates in steady state

Mouse strain	Organ	Percentage of all acquired cells					Absolute numbers (1.0e + 07)
		CD3^+^	CD4^+^	CD8^+^	CD19^+^	NKp46^+^	
WT	Spleen	20.6 ± 4.7	10.3 ± 2.7	8.20 ± 1.0	23.4 ± 6.7	0.92 ± 0.5	6.7 ± 1.4
vavFLIP_R_	Spleen	21.7 ± 3.5	11.6 ± 2.3	7.45 ± 1.5	22.7 ± 6.4	0.87 ± 0.3	6.3 ± 2.3
WT	Lymph node	33.9 ± 3.7	23.8 ± 2.5	16.0 ± 2.2	10.5 ± 1.1	0.21 ± 0.03	1.6 ± 0.6
vavFLIP_R_	Lymph node	34.1 ± 2.9	22.1 ± 2.0	17.7 ± 1.0	12.1 ± 1.6	0.19 ± 0.04	1.4 ± 1.3

Since the higher disease score suggested a higher susceptibility of vavFLIP_R_ mice to IAV infection, we quantified the viral load in the lungs of infected mice. For this purpose, mRNA copies of the IAV NP gene were measured. In the early phase of infection (days 1–2 p.i.), the number of NP copies between infected WT and vavFLIP_R_ mice was comparable (Fig. 1D). However, significantly higher NP gene expression was observed in vavFLIP_R_ mice on days 3, 5, and 7 p.i. (Fig. 1D), which reached a 15.1-fold difference on day 7. This was further confirmed by foci assays indicating replication-competent virus at day 7 p.i. (Fig. 1E). Here, vavFLIP_R_ mice had exhibited a threefold higher viral titer compared to WT controls. The lower fold difference in infectious virus compared to viral RNA copies might indicate that also unproductive virus is generated during infection. Notably, the viral load in vavFLIP_R_ and WT mice was below the level of detection at day 10 p.i. Furthermore, histological sections of the lungs from influenza-infected vavFLIP_R_ and WT mice were examined on day 7 p.i. Consistent with qPCR and foci assay data, large patches of virus-infected cells were detected in vavFLIP_R_ mice compared to WT mice (Fig. 1F). In conclusion, vavFLIP_R_ mice showed a higher viral titer at the peak of infection compared to WT littermates.

### *Increased NK cell accumulation in vavFLIP*_*R*_* mice during IAV infection*

**Fig. 2:**
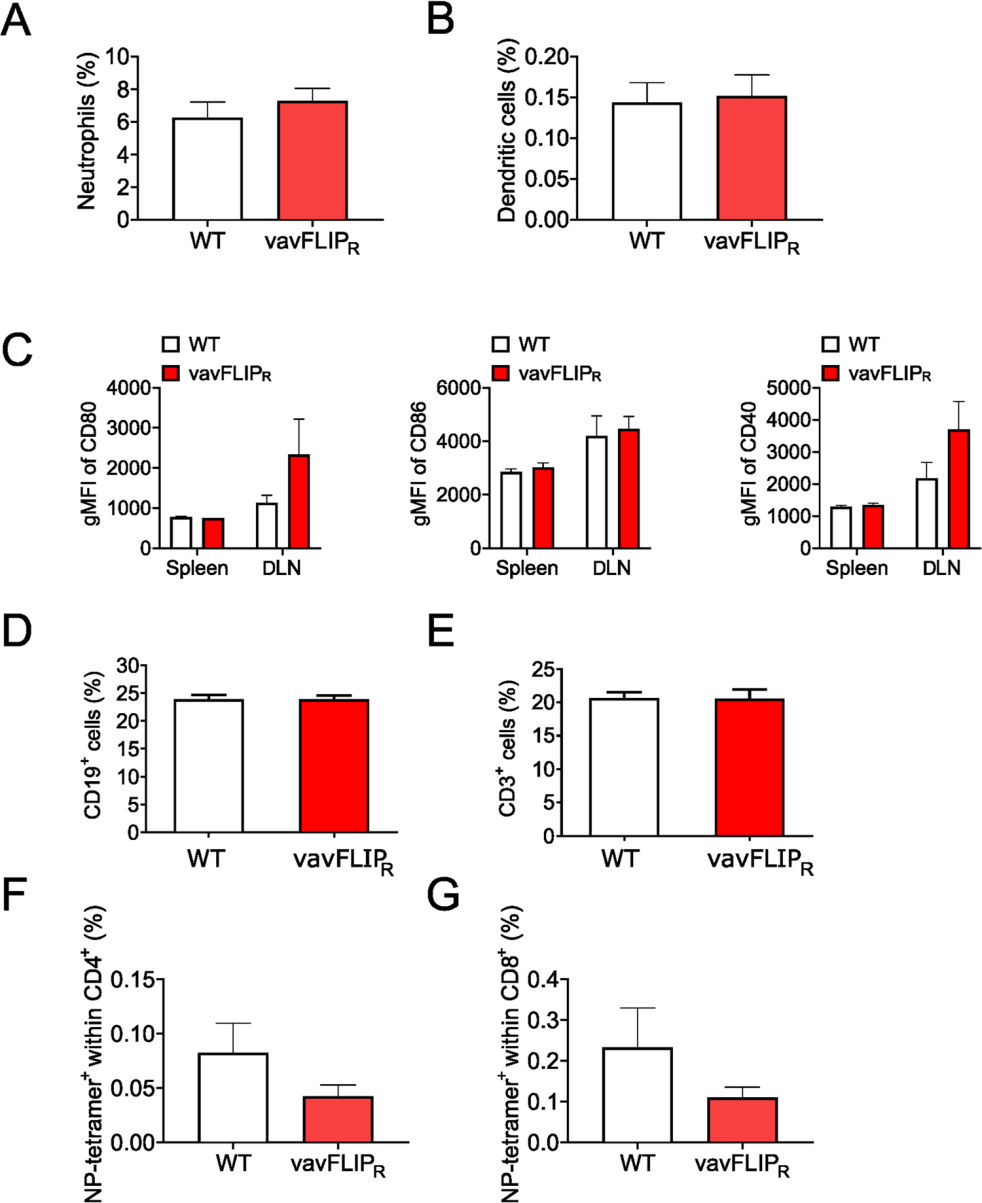
Responses of neutrophils, dendritic cells, and lymphocytes are only slightly altered in vavFLIP_R_ mice upon IAV infection. Single-cell suspensions from spleens of vavFLIP_R_ (red) and WT (white) mice were prepared on day 7 p.i. and stained for **A** Ly6G^+^ neutrophils; **B** CD11c^+^ dendritic cells; **C** the activation markers CD80 (left panel), CD86 (middle panel), and CD40 (right panel) expressed on CD11c^+^ dendritic cells; **D** CD19^+^ B cells; and **E** CD3^+^ T cells. **F-G** Antigen-specific T cells were identified by MHC tetramers loaded with a peptide derived from the influenza nucleoprotein (NP, aa 311–325) on CD3^+^CD4^+^ (F) and CD3^+^CD8^+^ T cells (G)

Our results suggested differences in innate immunity since the virus was eliminated in both genotypes at a time point when usually adaptive immunity is fully active. To investigate possible alterations in the cell type composition during the immune response following IAV infection, we analyzed lymphocytes by flow cytometry. We detected no significant differences in neutrophils (Ly6G^+^ cells) and dendritic cells (CD11c^+^ cells) (Fig. 2A, B). Despite a tendency for higher expression of CD40 and CD80 in dendritic cells from the draining lymph nodes of infected vavFLIP_R_ mice, the activation of dendritic cells was not significantly altered (Fig. 2C). While frequencies of total CD19^+^ B cells (Fig. 2D) and CD3^+^ T cells (Fig. 2E) were similar between vavFLIP_R_ mice and littermate controls, NP-reactive CD4^+^ and CD8^+^ T cells were slightly reduced in infected vavFLIP_R_ mice (Fig. 2F, G), consistent with reduced or slower activation of adaptive immunity by the innate immune system.

**Fig. 3:**
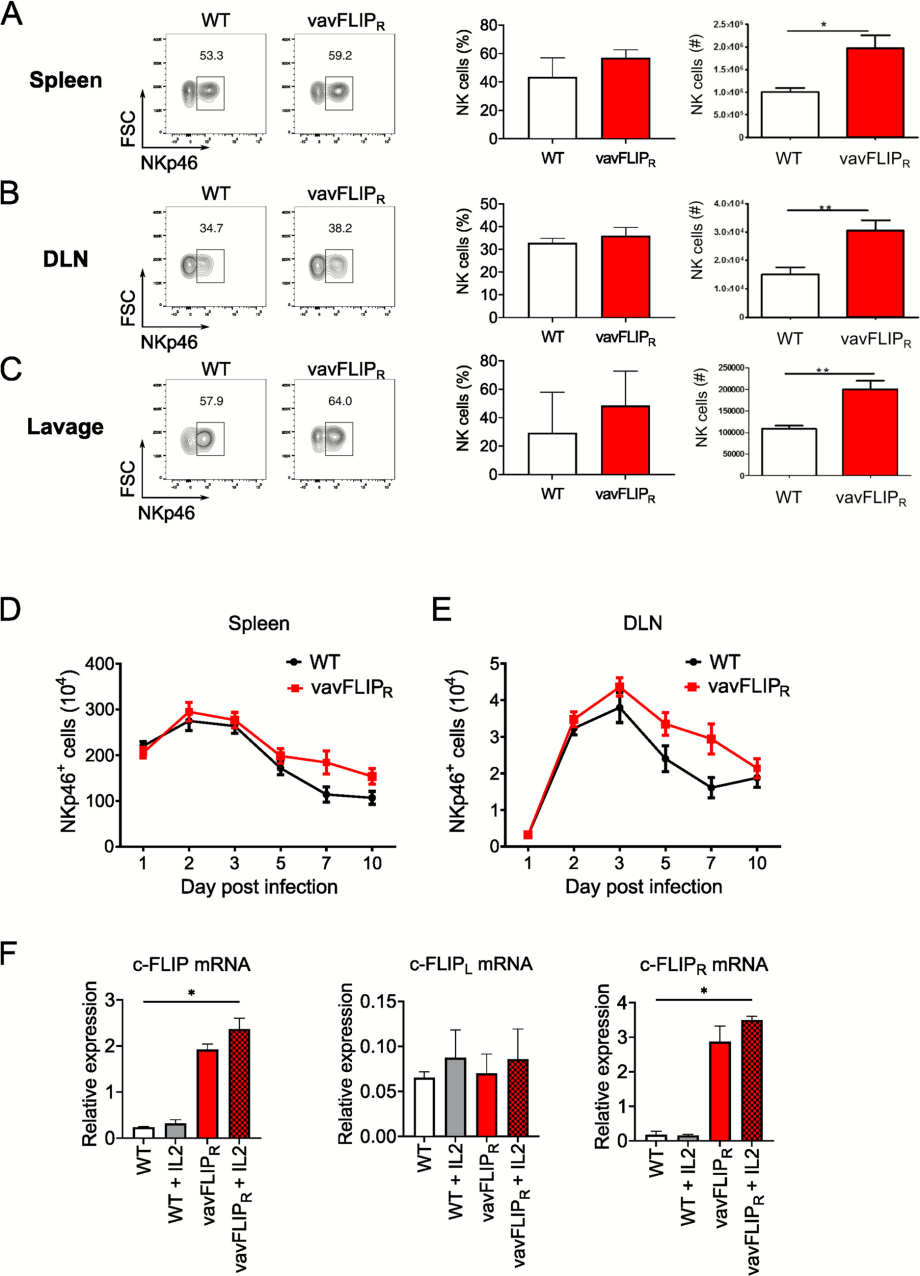
Higher number of NK cells at the peak of IAV infection in vavFLIP_R_ mice. **A-C** Single-cell suspensions from different organs of vavFLIP_R_ (red) and WT (white) mice were prepared on day 7 p.i. and stained for CD3, CD19, and NKp46. NK cell frequencies and cell numbers were determined from CD3 and CD19 double-negative cells. Representative dot plots are shown on the left of panels (A-C). Bar graphs represent the mean of frequencies and absolute numbers, respectively, from three independent experiments; error bars display SEM. Statistical analyses were performed with two-tailed nonparametric Mann–Whitney tests. *p < 0.05 and **p < 0.01. WT (*n* = 6), vavFLIP_R_ (*n* = 7). **A** NKp46^+^ cells from spleen, **B** NKp46^+^ cells from the mediastinal lymph nodes, and **C** NKp46^+^ cells from bronchoalveolar lavage. **D, E** Kinetics of NK cell accumulation during IAV infections. vavFLIP_R_ (*n* = 6) and WT (*n* = 6) mice were infected with IAV infection. NK cells were enumerated in the spleen (D) and the lung-draining lymph node (DLN) (E) at the indicated time points by NKp46 staining and flow cytometry. Shown are CD3^−^ CD19^−^ NKp46^+^ cells. **F** qPCR analysis of c-FLIP mRNA expression in purified NK cells stimulated with 50 ng/ml interleukin-2 for 3 h or left untreated. Shown is the expression of total c-FLIP mRNA (left panel), c-FLIP_long_ mRNA (middle panel), and c-FLIP_R_ mRNA (right panel). Statistical analyses were performed using Kruskal–Wallis one-way ANOVA (Dunn’s multiple comparison post-test); *p < 0.05

In contrast, the frequency and total numbers of NK cells were higher in the spleen (Fig. 3A), the mediastinal lymph node (Fig. 3B), and bronchoalveolar lavage fluid (Fig. 3C) of vavFLIP_R_ mice compared to WT littermates at the peak of infection. Furthermore, we analyzed the kinetics of NK cell recruitment in WT and vavFLIP_R_ mice in the spleen (Fig. 3D) and the lung-draining lymph node (Fig. 3E) by flow cytometry. Overall, both WT and vavFLIP_R_ mice showed comparable qualitative kinetics until day 10 p.i. (see Fig. 3D, E) with higher frequencies and numbers of NK cells in the analyzed compartments at day 7 p.i. in vavFLIP_R_ mice.

We have previously shown that c-FLIP expression in bone marrow NK cells is crucial for their development [29]. Therefore, we analyzed c-FLIP mRNA expression in mature splenic NK cells. As expected, vavFLIP_R_ mice expressed much more c-FLIP mRNA than control mice, which was mostly due to c-FLIP_R_ (Fig. 3F). Activation of NK cells by interleukin-2 slightly increased the expression of c-FLIP_long_ but had little effect on c-FLIP_R_ mRNA expression (Fig. 3F).

**Fig. 4:**
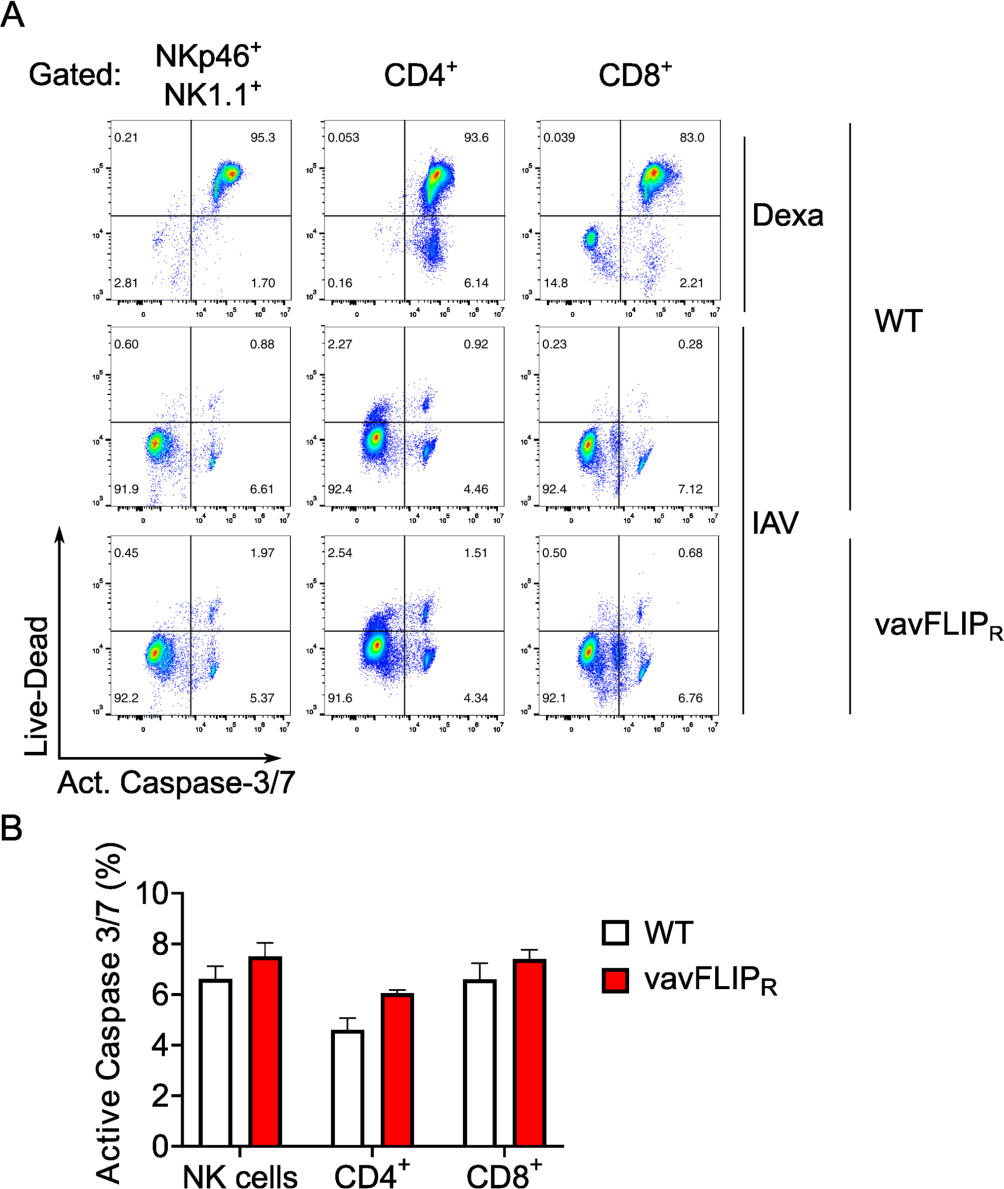
Immune cell death during IAV infection. **A** Representative flow cytometry dot plots of LIVE/Dead Fixable Blue and active caspase-3/7 in NKp46^+/^NK1.1^+^, CD8^+^, and CD4^+^ cells from the spleens of vavFLIP_R_ and WT mice on day 7 post-infection. Dexamethasone was used as a positive control for caspase activation and cell death on wild-type (WT) cells. **B** Active caspase-3/7 is displayed as the mean ± SEM from three independent experiments

Next, we analyzed whether alterations in lymphocyte apoptosis contributed to NK cell accumulation of IAV infected vavFLIP_R_ mice. To this end, NKp46^+^ NK cells, CD4^+^ T cells, and CD8^+^ CTLs from both genotypes were analyzed for the activity of the apoptosis executioners caspase-3 and caspase-7, and dead cells via flow cytometry. Dexamethasone efficiently killed all three lymphoid cell types (Fig. 4A). The frequency of cells with active caspase-3/7 in the CD4^+^, CD8^+^ T cell, and NK cell compartments was comparable in IAV-infected vavFLIP_R_ and WT mice (see Fig. 4A, B). This result suggests that c-FLIP_R_ expression in NK cells does not affect NK cell survival during IAV infection.

### *Depletion of NK cells blunts differences in viral load between WT and vavFLIP*_*R*_* mice*

**Fig. 5:**
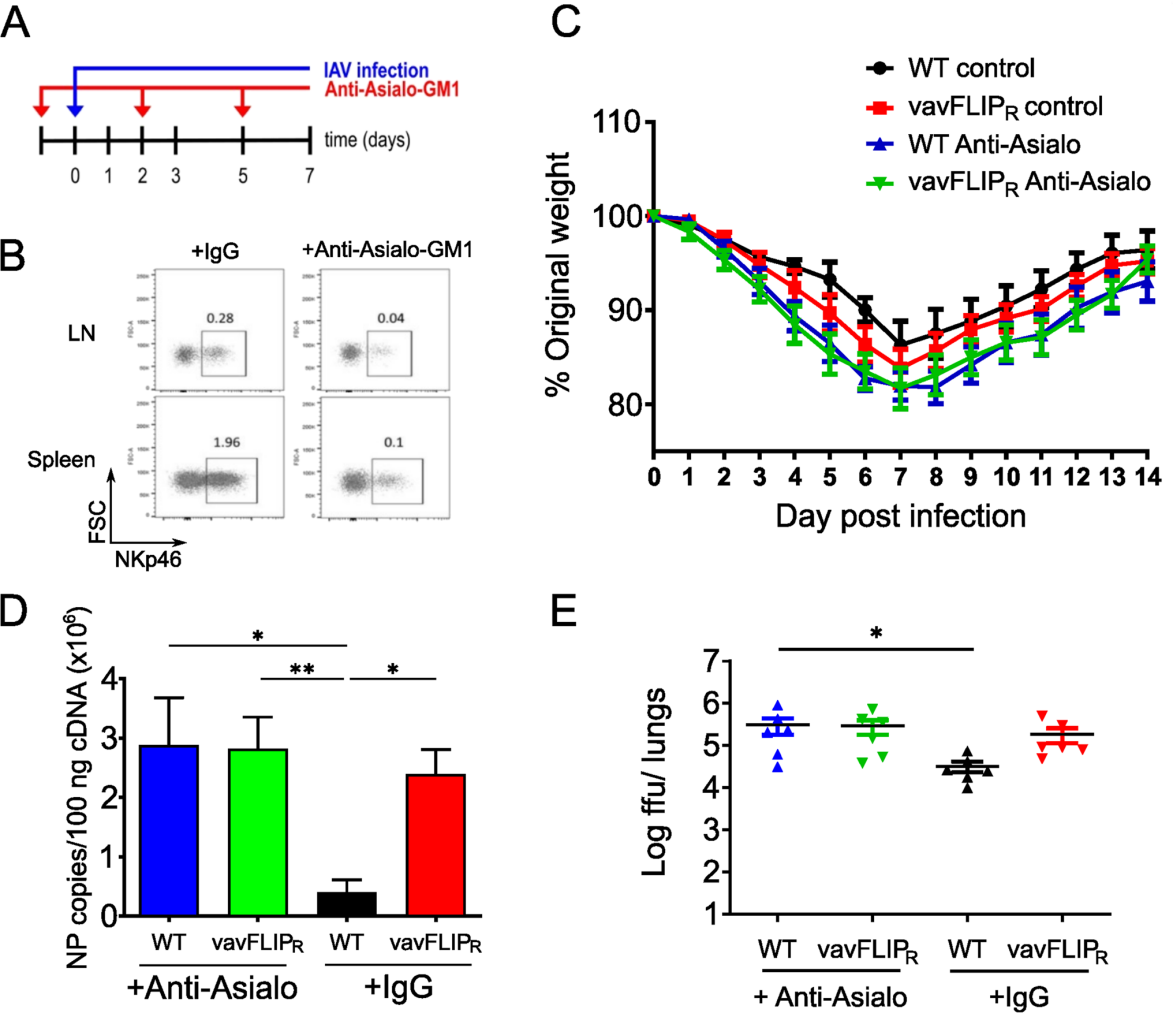
In vivo NK cell depletion with Anti-Asialo-GM1 abolishes differences in viral load between vavFLIP_R_ and control mice. **A** Mice were inoculated i.p. with 20 µg Anti-Asialo-GM1 antiserum (or IgG control), 1 day prior to infection and received another 20 µg on day 2 and 5 p.i. **B** Representative dot plots showing the efficiency of depletion at day 7 p.i. **C** Following infection, mice were monitored daily for body weight for 14 days. Data are presented as mean value ± SEM. *n* = 5 per group of mice. **D, E** NK cell-depleted and control mice were infected with influenza A virus. Subsequently, virus titers were determined by **D** qPCR and **E** foci assay in the lungs of vavFLIP_R_ and WT littermates on day 7 post-infection. Bar graphs represent the mean; error bars represent SEM. Statistical analyses were performed using Kruskal–Wallis one-way ANOVA (Dunn’s multiple comparison post-test); *p < 0.05 and **p < 0.01

To investigate the influence of a higher number of NK cells during IAV infection, mice were treated with Anti-Asialo-GM1 to deplete NK cells or a control IgG in vivo prior to and during influenza infection (Fig. 5A). Anti-Asialo-GM1 was reported effective at the depletion of NK cells in vivo [30, 31], and did so in both vavFLIP_R_ and WT mice as confirmed by flow cytometric analysis of splenocytes (Fig. 5B). The body weight of the NK cell-depleted mice was recorded daily for 14 days post-infection. Similar to NK cell-proficient mice, no significant difference in body weight loss in NK cell-depleted vavFLIP_R_ and NK cell-depleted WT mice was observed (Fig. 5C). Next, mice depleted of NK cells were infected with IAV, and the viral load was measured on day 7 p.i. by qPCR and foci assay. As expected, control IgG-treated vavFLIP_R_ mice showed a significantly higher viral load on day 7 compared to WT control mice (Fig. 5D, E). NK cell depletion resulted in a higher viral load in both vavFLIP_R_ and WT mice compared to controls injected with control rabbit IgG (Fig. 5D, E), consistent with the notion that NK cells are important to combat viral infections. However, the differences in viral load between vavFLIP_R_ and WT mice disappeared following NK cell depletion, suggesting that the increased NK cell numbers are mechanistically linked to a higher viral load in vavFLIP_R_ mice. In order to test this hypothesis, we further characterized the NK cell response in vavFLIP_R_ mice during IAV infection.

### IAV infects primary mouse NK cells

**Fig. 6:**
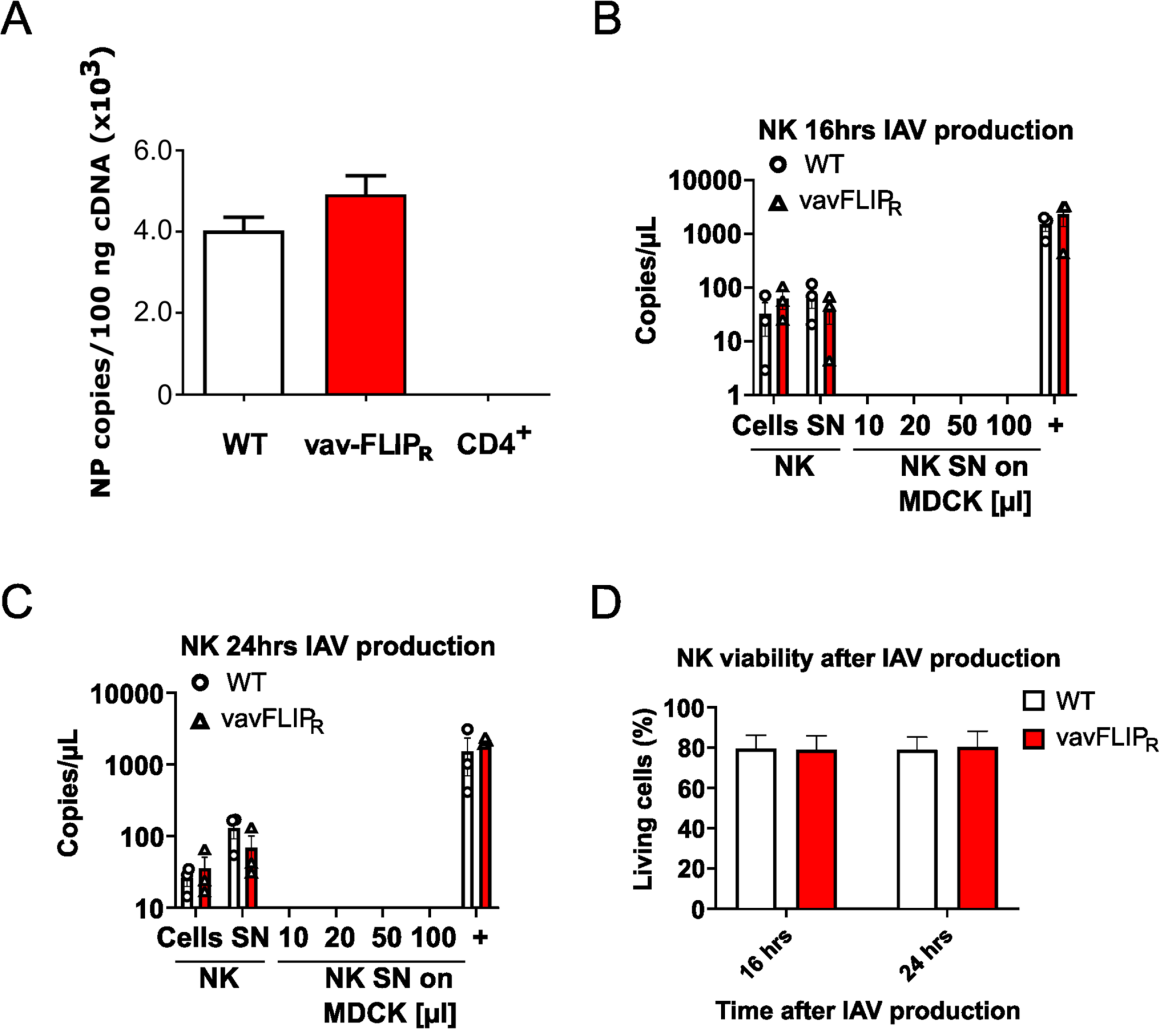
Influenza virus directly infects primary murine NK cells. **A** Splenic NK cells from vavFLIP_R_ mice and WT littermates were purified by magnetic-activated cell sorting (MACS) and incubated with influenza virus H1N1 at a multiplicity of infection (MOI) of 2. After 1 h of viral adsorption, unadsorbed viruses were washed away by excess phosphate buffered saline. CD4^+^ cells were treated in parallel as a negative control. The expression of viral NP mRNA was detected by qPCR after an additional incubation time of 4 h. **B, C** NK cells from vavFLIP_R_ mice and WT littermates were isolated by MACS, stimulated with IL-2 and IL-15, and infected with influenza virus H1N1 at a multiplicity of infection (MOI) of 2 for 2 h. After extensive washing, NK cells were cultured for 16 h (B) and 24 h (C). Additionally, supernatants of the infected NK cells were collected and 10, 20, 50 and 100 l of the supernatants were transferred onto MDCK cells and incubated for 24 h. RNA was purified from cells as well as NK cell supernatant and tested by qPCR for NP mRNA expression. Directly infected MDCK cells (+) served as positive control. **D** The viability of NK cells during infection was measured by 7-AAD staining

Since it was reported that IAV can infect NK cells [32–34], we asked whether c-FLIP_R_ affects the infection of NK cells. To test this experimentally, freshly isolated primary NK cells were infected with H1N1 influenza virus and subsequently washed and cultured. Using RT-PCR, the NP gene was detected in IAV-infected NK cells (Fig. 6A). In contrast, no NP gene was detected in IAV-exposed CD4^+^ T cells (Fig. 6A), which is in line with a report that primary human CD4^+^ T cells cannot be infected by IAV [35]. Next, we addressed the question of whether or not IAV infection of NK cells results in productive infection. To this end, NK cells from vavFLIP_R_ and WT mice were activated by IL-2 and IL-15, infected for 2 h, washed extensively, and incubated with fresh medium for an additional 16 and 24 h. Subsequently, the supernatants were inoculated onto MDCK cells. Twenty-four hours later, qPCR for NP RNA expression was performed to determine if infectious progeny were produced in MDCK cells. We detected similar levels of NP RNA in IAV-treated NK cells and within supernatants from NK cells at 16 and 24 h post infection, indicating that IAV is able to infect and replicate in primary NK cells (Fig. 6B, C). However, viral copy numbers were much lower than in infected MDCK cells. Importantly, the transfer of supernatants of infected NK cells onto MDCK cells did not result in infection (Fig. 6B, C), implying that NK cells are not productively infected by IAV. In addition, infection with IAV did not result in a substantial loss in viability of NK cells (Fig. 6D). Taken together, our results suggest that IAV does probably not lead to a productive, lytic infection of NK cells and that the increased viral titers might be caused by functional impairment of these cytotoxic lymphocytes.

### *Degranulation and cytokine expression is impaired in NK cells of vavFLIP*_*R*_* mice during IAV infection*

**Fig. 7:**
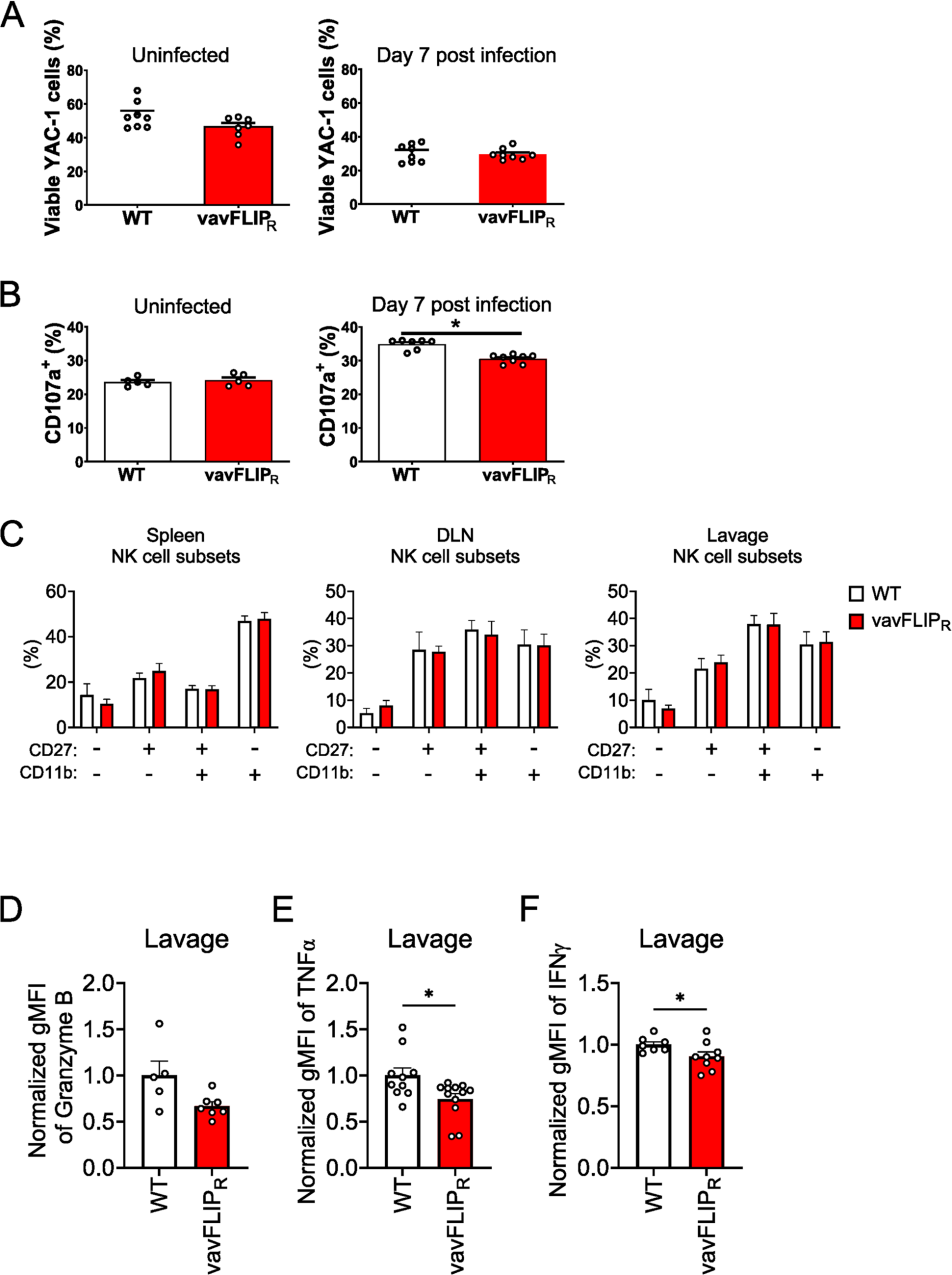
IAV infection impairs NK cell functionality. **A** NK cells of uninfected (left panel) and IAV-infected (right panel) vavFLIP_R_ mice and WT littermates were purified by flow cytometry and incubated with YAC-1 cells at a ratio of 1:8 for 16 h. After the incubation time, cells were harvested and analyzed by flow cytometry. **B** Flow cytometry-purified NK cells from spleens of uninfected (left panel) and 7-day-infected (right panel) vavFLIP_R_ mice and WT littermates were incubated with YAC-1 cells at an effector-to-target ratio of 1:1 in the presence of brefeldin A, monensin, and CD107a antibody for 2 h. Surface expression of CD107a on the uninfected and infected NK cells of vavFLIP_R_ mice and WT littermates was analyzed by flow cytometry using anti-CD107a antibody. **C** Bar graphs showing the percentage of different maturation stages of NK cells according to CD11b and CD27 expression in pregated NK cells of spleen (left panel), lung-draining lymph nodes (middle panel) and bronchoalveolar lavage (right panel) from vavFLIP_R_ and WT mice at 7 days post IAV infection. NK cells were pre-gated with forward and side scatter to remove debris, gated on single cells, living cells with LIVE/DEAD™ fixable blue dead cell stain kit and on CD3^−^ CD19^−^ NKp46^+^ NK1.1^+^. **D** Bar graph showing the mean fluorescence intensity (MFI) of granzyme B of cells from bronchoalveolar lavage of vavFLIP_R_ and WT mice at 7 days post IAV infection. NK cells were pre-gated with forward and side scatter to remove debris, gated on single cells, living cells with LIVE/DEAD™ fixable blue dead cell stain kit and on CD3^−^ CD19^−^ NKp46^+^. **E** Bar graph showing the mean fluorescence intensity (MFI) of TNFα of cells as in (D). **F** Bar graph showing the mean fluorescence intensity (MFI) of IFNγ of cells as in (D). Each dot represents an individual mouse in the graphs. Bar graphs show mean ± SEM. Statistical analyses were performed using two-tailed Mann–Whitney tests; *p < 0.05

In order to characterize the functionality of NK cells from vavFLIP_R_ and WT mice, we first did a standard cytotoxicity assay with YAC-1 target cells. The results indicated that the cytotoxicity of NK cells from vavFLIP_R_ and WT mice was comparable under these in vitro conditions (Fig. 7A). In addition, the activity of NK cells was measured via cell surface exposure of CD107a during IAV infection. CD107a is localized to lysosomes and cytolytic granules in resting NK cells [36, 37]. Upon activation, NK cells degranulate their cytolytic contents and CD107a appears on the NK cell surface. Therefore, the degranulation of NK cells following interaction with IAV-infected cells was analyzed by the cell surface expression level of CD107a in vavFLIP_R_ and WT mice during the course of IAV infection via flow cytometry. Percentages of CD107a-positive cells were significantly lower in the infected vavFLIP_R_ mice compared to WT littermates (Fig. 7B). Next, immune phenotyping of NK cells during IAV infection was performed. We stained for CD11b, CD27, granzyme B, interferon-γ, KLRG1, and TNFα in splenic NK cells at day 7 p.i. We found no significant differences in the frequencies of NK cell maturation stages according to CD11b and CD27 expression (Fig. 7C). Moreover, the frequency of positive cells or mean fluorescence intensity of KLRG1 was not altered in spleen, draining lymph node, and lung lavage (data not shown). The expression of granzyme B, interferon-γ and TNFα on a per cell basis was reduced in cells from lung lavage (Fig. 7D–F). Taken together, these results suggest that the cytotoxic activity of NK cells and their ability to communicate with adaptive immune cells might be impaired in vavFLIP_R_ mice during IAV infection in vivo.

## Discussion

Programmed cell death plays an important role in host defense mechanisms to eliminate infected cells, reducing the spread of infection to neighboring cells and preventing pathogen persistence. During IAV infection, apoptosis and necroptosis are induced in infected host cells [15, 16, 18]. Notably, apoptosis has to be tightly regulated since too little or too much cell death may lead to pathology [38].

In order to study the regulation of the extrinsic pathway of apoptosis by c-FLIP_R_, vavFLIP_R_ transgenic mice were utilized that constitutively express murine c-FLIP_R_ in all hematopoietic cells. As described before [8], normal numbers of immune cells in the steady state were observed in vavFLIP_R_ mice. When challenged with *Listeria monocytogenes*, vavFLIP_R_ mice exhibited better bacterial clearance [8]. In contrast, we showed herein that when challenged with IAV, vavFLIP_R_ presented a more severe acute infection compared to WT, characterized by slightly increased weight loss and significantly higher viral load around the peak of infection. No significant differences were observed in myeloid cells, as well as B and T cells in infected vavFLIP_R_ versus WT mice, although there was a slight increase in the expression of certain activation markers on dendritic cells. However, we also detected slightly lower frequencies of antigen-specific CD4^+^ and CD8^+^ T cells. Importantly, total NK cell numbers were significantly higher in the lung-draining lymph node of infected vavFLIP_R_ mice, implicating a role of innate immunity in the exacerbated viral load kinetics. Therefore, the role of NK cells in the severity of IAV infection in vavFLIP_R_ mice was further assessed in vivo and in vitro.

NK cells play a pivotal role in the immune response against IAV infection. However, the exact role of NK cells in the immune response against different virus families is not fully understood. Several studies have pointed out the importance of NK cells in the control of IAV infection, in which depletion of NK cells or defects in NK cell activity can cause morbidity and mortality or delayed viral clearance [39, 40]. In contrast, some studies illustrated that NK cells exacerbate the pathology of the IAV infection in mice [27, 41, 42]. We assessed the impact of the higher number of NK cells on host outcome during IAV infection by treating vavFLIP_R_ and WT mice with anti-Asialo-GM1 to deplete NK cells in vivo before and during IAV infection. NK cell depletion in both vavFLIP_R_ and WT mice resulted in an increased viral load at the peak of infection compared to the control mice. However, the differences between vavFLIP_R_ and WT in the viral load were resolved by NK cell depletion, suggesting that an increased number of NK cells in IAV infection may have an unfavorable outcome for the host. Zhou et al. reported that depletion of NK cells ameliorates the survival rate in high-dose, but not medium- or low-dose influenza infection [27]. Similarly, Waggoner et al. showed that virus dosage can affect the immuno-regulatory function of NK cells [43]. Therefore, comparing the kinetics of NK cells in high and low doses of influenza infections might be helpful in better understanding the opposing roles of NK cells in IAV infections. Consistent with previous reports [32–34], we demonstrated that IAV can directly infect and replicate within primary murine NK cells, although viral replication within NK cells was not productive. Nevertheless, IAV infection of NK cells might be a cause of impaired NK cell functionality.

Although the cytotoxicity of NK cells from vavFLIP_R_ mice against a standard tumor cell line as target was not significantly altered compared to WT controls, ex vivo analysis of NK cell degranulation from IAV-infected mice showed a significant reduction in degranulation in c-FLIP_R_-expressing NK cells. Moreover, we detected reduced effector cytokine expression and a tendency of lower granzyme B expression in NK cells from the lung lavage of vavFLIP_R_ mice. Upon NK cell stimulation, the activation signal mediates a downstream cascade of kinase activation, which results in exocytosis of cytotoxic granules and subsequently kills the target cells [44–46]. On the other hand, IAV has developed evasion strategies, which can enable the virus to impair the effector functions of NK cells. As a part of immune evasion strategies, the virus can directly infect NK cells and induce cell apoptosis [34]. Direct infection of NK cells by IAV was also shown to interfere with the ability of NK cells to effectively recognize and kill the target cells [32, 33]. Moreover, IAV infection of NK cells decreased the cytotoxic potential of cytotoxic T cells [33].

Surprisingly, c-FLIP_R_-expressing NK cells were not protected from cell death during IAV infection. Thus, c-FLIP_R_ might act in a non-cell death manner in NK cells, which is different from its role in T cells during Listeria infection [8]. So, what could be a potential mechanism of c-FLIP inhibiting NK cell function? The transcription factor IRF3 is important for innate immune responses against viruses, including NK cell function [47–49]. For instance, it is crucial for granzyme B and IFNγ expression in NK cells [48]. Interestingly, c-FLIP has been shown to inhibit IRF activity by direct binding and independent of IRF3 degradation or dephosphorylation [50, 51]. Although this was specific for c-FLIP_long_, the splice variant that contains a caspase-like domain and nuclear localization sequences [50], it is conceivable that the higher expression of c-FLIP_R_ in vavFLIP_R_ mice liberates endogenous c-FLIP_long_ from canonical anti-apoptotic functions, allowing it to inhibit IRF3 in the nucleus of NK cells. We conclude that c-FLIP_R_ expression in NK cells may lead to impaired function during infection, which in turn leads to IAV being able to infect lung epithelial cells more efficiently. Therefore, IAV titers are increased in vavFLIP_R_ mice in the early phase of the immune response. IAV titers are normalized upon induction of the adaptive immune response between day 7 and day 10.

In summary, we presented a combined in vivo and in vitro approach that provided supporting evidence that NK cells may play a pathogenic role during IAV infections. However, the contribution of NK cells to the protective response against IAV is indispensable despite being exploited by the virus for replication. Our findings underline the importance of strict regulation of apoptosis in limiting the escape strategy of viruses and establishing antiviral immunity.

## Data Availability

The datasets generated during the current study are not publicly available but are available from the corresponding author on reasonable request.
